# The dynamic world of the 8–17 DNAzyme

**DOI:** 10.1038/s41467-024-49500-w

**Published:** 2024-06-17

**Authors:** Jessica Felice Schmuck, Jan Borggräfe, Manuel Etzkorn

**Affiliations:** 1 Heinrich Heine University, Faculty of Mathematics and Natural Sciences, Institute of Physical Biology, Düsseldorf, Germany; 2 Institute of Structural Biology, Molecular Targets and Therapeutics Center, Helmholtz Zentrum München, Neuherberg, 85764 München, Germany; 3 Bavarian NMR Center, School of Natural Sciences, Technical University of Munich Garching, 85748 München, Germany; 4 Institute of Biological Information Processing (IBI-7), Forschungszentrum Jülich, Jülich, Germany

**Keywords:** Solution-state NMR, Catalytic DNA, Enzymes

## Abstract

DNA catalysts, known as DNAzymes, have arguably been limited for decades by the lack of mechanistic information. The solution structure of the 8–17 DNAzyme reported by Wieruszekska, Pwlowicz et al. reassesses the current thinking regarding the relationship between structure, dynamic, and metal ion coordination.

High-precision DNA-based biocatalysts, known as DNA enzymes or DNAzymes, are experiencing a revival in biotechnological, analytical, and therapeutical applications. Discovered in the 1990s, mechanistic insights in their mode-of-action have been sparse for decades, coinciding with inherent limitations associated with their desired cellular applications. It appears that the current emerging interest in the DNAzyme technology is primarily due to three technological advances: (i) general progress in related nucleic acid-based therapeutics overcoming limitations in DNAzyme delivery, (ii) the availability of chemically modified building blocks to increase bioactivity and stability, and (iii) developments in our mechanistic understanding of the system. The latter gained momentum by deciphering several molecular structures of DNAzymes; starting with the crystal structure of the RNA-ligating 9DB1 DNAzyme^1^, followed by the crystal structure of the 8–17 DNAzyme^2^, and the recent solution structure of the 10-23 DNAzyme^3^. The 8–17 and 10-23 DNAzymes are among the most prominent DNAzymes and are capable of target-selective RNA cleavage^4^.

The work of Wieruszekska, Pwlowicz et al. now contributes the solution structure of the 8–17 DNAzyme (Dz)^5^. A remarkable feature of the structure (solved in presence of Zn^2+^ and Na^+^) is its high similarity to the corresponding crystal structure (solved in presence of Pb^2+^). This result reshapes the current understanding of the Dz mechanism, so far suggested to use different structural arrangements associated with the coordination of different metal ions. To obtain the reported Dz solution structure, Wieruszewska, Pawlowicz et al. optimized construct design to improve NMR-spectral features (Fig. 1a, b). Generating a single-sequence DNA construct with shortened arms (Fig. 1b) yielded well-resolved data and enabled structure calculation of an NMR ensemble with low RMSD values (Fig. 1c, d).

**Fig. 1:**
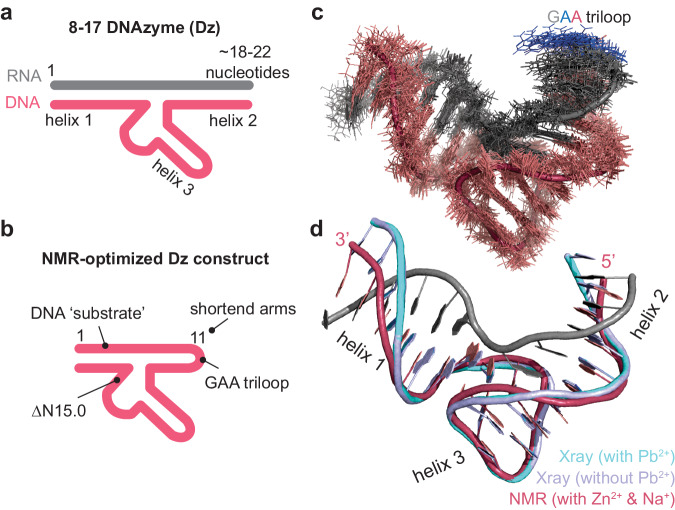
Overview of the 8–17 DNAzyme (Dz) system. Schematics of the general Dz design (**a**) and the construct designed for NMR studies (**b**). **c** NMR ensemble (20 structures) determined by Wieruszekska, Pwlowicz et al.^5^ using the construct shown in (**b**). For simplicity the substrate sequence of the all-DNA construct is shown in grey and the GAA-triloop in indicated colours. **d** Comparison of recently reported solution structure (pink, pdb code: 8OR8) in presence of Zn^2+^ and Na^+^, with previous crystallographic data obtained in the presence (cyan) or absence of Pb^2+^ (purple)^2^, pdb code: 5XM8 and 5XM9, respectively. For simplicity the substrate strand is only shown for the NMR structure (grey) and the triloop is omitted.

A central aspect of DNAzyme activity is the role of metal-ion cofactors, essential for Dz-mediated substrate cleavage. Previous studies have demonstrated that different divalent metal ions can serve as co-factors. The solution structure of Dz was determined in the presence of Zn^2+^ and Na^+^ and is structurally similar to the crystal structures of Dz in the absence and presence of Pb^2+^^2^, this finding is surprising since previous FRET studies showed, that the Zn^2+^ and Mg^2+^ bound forms of Dz are more condensed than the metal-ion-free state^6,7^. In addition, a condensation step was also observed preceding the metal-ion-induced substrate cleavage^6^. In contrast this effect could not be observed for the catalytically more activating Pb^2+^, which led to the long-standing hypothesis that Pb^2+^-dependent catalysis follows a different mechanism dictated by a distinct fold^6^.

To better understand and explain the potentially contradicting results Wieruszewska, Pawlowicz et al. studied the Zn^2+^, Mg^2+^, Na^+,^ and Pb^2+^ induced folding by NMR and CD spectroscopy^5^. The respective titration experiments on non-cleavable (all DNA) constructs revealed comparable structural effects induced by all tested metal ions. However, a substantial dissimilarity between Pb^2+^ and the other tested divalent ions was detected, when the titration was complemented with activity assays. Here the observed rate constants of Mg^2+^ and Zn^2+^ induced substrate cleavage (k_obs_) reached saturation in the mM range. This is about two orders of magnitude larger that the observed apparent dissociation constant (K_d_) of the respective metal ions that is associated with the Mg^2+^/Zn^2+^-induced structural changes. This observation suggests the presence of at least two metal ion binding side (MBS I and II), one that induces structural condensation (MBS I with high affinity for Mg^2+^ and Zn^2+^) and a second binding side additionally required for substrate cleavage (MBS II with lower affinity for Mg^2+^ and Zn^2+^). In strong contrast for Pb^2+^ titrations, k_obs_ is saturated at much lower Pb^2+^ concentrations, which are comparable to the respective K_d_ values for Pb^2+^ binding to MBS I.

Based on their observations Wieruszekska, Pwlowicz et al. develop a unified model that can accommodate the previous and current data. According to this model the interplay of dynamics and conformational plasticity and its modulation by the respective metal ions is the central element of Dz activity (Fig. 2). In this regard, the differential affinities of Mg^2+^/Zn^2+^ to MBS I and II will promote the population of an activated precatalytic complex that is characterized by a structural condensation induced by occupancy of MBS I. Subsequent and less frequent occupation of MBS II will than lead to substrate cleavage. The presence of Pb^2+^, however, does not lead to a strongly overpopulated condensed form, since comparable affinities to MBS I and II could promote cleavage before stabilizing a condensed form over a longer time. Remaining differences in reported behaviours could then be attributed to blind spots in the respective detection techniques, variations in experimental conditions, or differential behaviour associated with different sequences. To further support their model, Wieruszekska, Pwlowicz et al. also pinpoint MBS I via chemical shift perturbations, showing that MBS I is not directly involved in catalysis, and demonstrating that scaffolding the Dz with high concentrations of Mg^2+^ assists cleavage in presence of Pb^2+^^5^. In light of this model, it can be argued that the solution structure is in its activated condensed form, since all ion-dependent effects are saturated under the applied conditions (i.e., 3 mM Zn^2+^, 200 mM NaCl). The high similarities to the previous crystal structures of the Dz’s Pb^2+^-bound form and in absence of Pb^2+^ (Fig, 1d), further suggest that the respective structures represent the same state and may have formed due to selection bias under the chosen crystallization conditions.

**Fig. 2:**
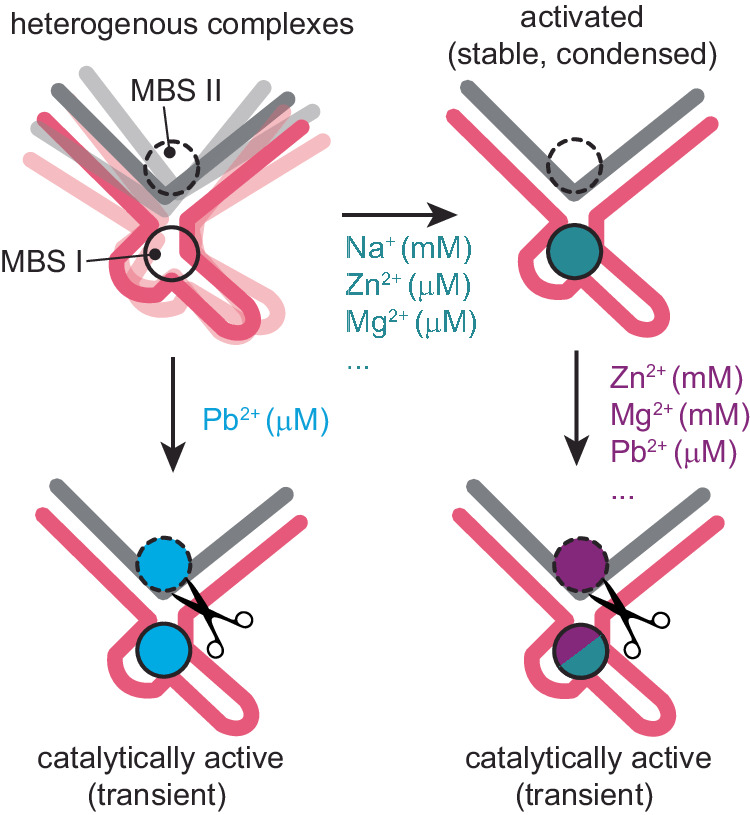
Unifying model proposed by Wieruszekska, Pwlowicz et al. to explain the effect of different metal ion on Dz’s structure and activity. According to this model the DNA’s catalytic capability is associated with at least two metal ion binding sides (MBS I and II). Binding in MBS I will induce a condensed state. (Additional) binding in MBS II will promote the cleavage reaction itself. Different metal ions will have differential affinities to the two MBSs (indicated by molarity values in parenthesis). Among the tested metal ions, Pb^2+^ has by far the highest affinity for MBS II. As a consequence, the presence of Pb^2+^ will directly promote cleavage without considerably increasing the population of the condensed (activated) state.

## Concluding Remarks

In general, the structural data reported by Wieruszekska, Pwlowicz et al. confirm that NMR spectroscopy is a powerful tool to determine a well-defined molecular arrangement of DNAzymes in solution (Fig. 1c). Simultaneously, these data also identify the interplay of multiple conformational, dynamic exchange processes, conformational plasticity, and its modulation by metal ions as central features responsible for the catalytic capability of the system. Excitingly, these features share high similarities with the 10-23 DNAzyme despite their strongly diverging structural architecture^3,8^, suggesting that DNA catalysts are highly dynamic molecules that are exceptionally dependent on the applied measurement condition. In this respect the strength of NMR spectroscopy is the ability to investigate the system in solution, and under adequate buffer conditions and temperatures. However, determining the exact localization of the central metal ion cofactors is still a bottleneck of the technique and may strongly benefit from complementary crystallographic and molecular dynamic simulations capturing the catalytically relevant state. The dynamic features of the system as well as a quantitative analysis of the occurrence and population of different states may be sensitive to construct engineering and crystallisation conditions. Therefore, these aspects must be carefully evaluated when linking the respective structural features with their mechanistic implications in the native system.

The structural and biophysical data reported by Wieruszekska, Pwlowicz et al. unravel key features of the system while highlighting a number of open questions that should be investigated in subsequent studies. These include the importance of buffer conditions to disentangle the effects of monovalent and divalent metal ions and potential cooperative effects between multiple binding sites in FRET studies, as well as possible effects of different arm sequences and artificial modifications.

In conclusion, it appears that a better understanding of the dynamic world of DNA catalysts is emerging as a central element in our view of these fascinating systems. In this picture specific DNA sequences, which on their own predominantly sample catalytically inactive conformations, can be stabilized in an active conformation by the appropriate metal ions. While this scaffolding step may be required for activity, it may not be sufficient, advocating for the need of a second metal ion for catalysis. Consequently, metal-ion dependent affinities, occupation times in the different binding sides, and cooperative effects determine the population of activated (scaffolded) states as well as catalytic rates. For the 8–17 DNAzyme, the data suggest that Pb^2+^ is a particularly potent cofactor due to its favourable interactions in MBS II that are essential for catalysis.
